# Concurrent loss of ciliary genes *WDR93* and *CFAP46* in phylogenetically distant birds

**DOI:** 10.1098/rsos.230801

**Published:** 2023-08-23

**Authors:** Buddhabhushan Girish Salve, Amia Miriam Kurian, Nagarjun Vijay

**Affiliations:** Computational Evolutionary Genomics Lab, Department of Biological Sciences, IISER Bhopal, Bhauri, Madhya Pradesh, India

**Keywords:** cilia, respiratory epithelium, segmental deletion, influenza, central apparatus, zoonosis

## Abstract

The respiratory system is the primary route of infection for many contagious pathogens. Mucociliary clearance of inhaled pathogens is an important innate defence mechanism sustained by the rhythmic movement of epithelial cilia. To counter host defences, viral pathogens target epithelial cells and cilia. For instance, the avian influenza virus that targets ciliated cells modulates the expression of *WDR93*, a central ciliary apparatus C1d projection component. Lineage-specific prevalence of such host defence genes results in differential susceptibility. In this study, the comparative analysis of approximately 500 vertebrate genomes from seven taxonomic classes spanning 73 orders confirms the widespread conservation of *WDR93* across these different vertebrate groups. However, we established loss of the *WDR93* in landfowl, geese and other phylogenetically independent bird species due to gene-disrupting changes. The lack of *WDR93* transcripts in species with gene loss in contrast to its expression in species with an intact gene confirms gene loss. Notably, species with *WDR93* loss have concurrently lost another C1d component, *CFAP46*, through large segmental deletions. Understanding the consequences of such gene loss may provide insight into their role in host–pathogen interactions and benefit global pathogen surveillance efforts by prioritizing species missing host defence genes and identifying putative zoonotic reservoirs.

## 1. Introduction

The respiratory epithelium is the first line of defence that acts as a physico-chemical barrier through antimicrobial host defence molecules and actively performs mucociliary clearance of inhaled pathogens [1–3]. The epithelium of the airway mostly consists of ciliated, goblet and basal cells, along with several other rarer cell types such as club cells, deuterosomal cells, ionocytes, neuroendocrine cells and tuft cells [4]. Adjacent epithelial cells fuse to form cell–cell junctions, which regulate the movement of molecules and physically impede viral entry into the submucosa [5,6]. Additionally, pattern recognition receptors (PRRs) in the epithelium initiate the innate immune response against pathogens [7,8]. Hence, the airway epithelium provides multiple host defence factors against diverse pathogens [9–11].

Several viral pathogens, such as influenza virus, rhinovirus, metapneumovirus, coronavirus (including SARS-CoV-2), respiratory syncytial virus and some bacterial pathogens, primarily infect the host through the respiratory route. Respiratory pathogens cause ciliary impairments such as ciliated epithelial cell shedding, ciliary motility disruption, loss of cilia, other types of ciliary dysfunction and ciliogenesis suppression [12–16]. Viruses can enter host cells by recognizing various sialic acid receptors of airway epithelium cells [17,18]. The human influenza virus infects cells with α-2,6-linked sialic acid receptors in contrast to avian influenza viruses that infect cells with α-2,3-linked sialic acid receptors [19]. Variation in sialic acid receptors has been linked to viral tropism, and a better understanding of their prevalence and tissue-specificity across diverse species may help understand zoonotic virus spillover [20–25]. For instance, a wider range of sialic acid receptor subtypes in chickens compared with ducks may cause infections from a broader range of avian influenza viruses [26]. Adaptation to differing pathogen regimes through the species-specific presence of sialic acids occurs through changes in the gene repertoire of sialic acid-converting enzymes [27,28]. Hence, gene presence/absence patterns in diverse vertebrate species could be associated with differences in the immune response [29–32]. Moreover, identifying natural knockouts of crucial genes can help uncover functional roles that could translate into biomedical insights and anticipate zoonotic events [33].

A transcriptomic study of the differential host response to high or low pathogenic H5N1 avian influenza virus in ducks has identified *WDR93* (also known as *CFAP297*) to be down-regulated in high and up-regulated in low pathogenic virus infection [34]. The *WDR93* gene has been predicted to be part of mitochondrial respiratory chain complex I due to its sequence homology with the *NDUFS4* gene. However, recent studies have conclusively established *WDR93* is part of the central apparatus projection D (C1d) along with *CFAP54*, *CFAP46* (also known as *TTC40*), *CFAP221* (also known as *PCDP1*) and *CAM1* [35–42]. Electron microscopy and gene-knockout studies have established the importance of the C1d projection in maintaining ciliary motility [36,43,44]. Single-cell transcriptomics of diverse non-model vertebrate species, including duck, has identified *WDR93* expressed in the ciliated cells of the lungs [45]. Hence, based on earlier literature, we reasoned that the *WDR93* gene expression change in response to H5N1 avian influenza in ducks might be a consequence of the virus targeting the ciliary apparatus [13]. Genome-wide RNA interference studies have also implicated *WDR93* in altering the percentage of cells infected with *M. tuberculosis* [14,46]. The interaction between the host gene *WDR93* with multiple pathogens hints at the possibility of lineage-specific gene losses that could alter host–pathogen interaction. In this study, we perform an in-depth comparative genomic analysis of the *WDR93* gene across diverse vertebrate lineages to evaluate gene presence/absence patterns. Ciliary assembly genes are down-regulated upon infection by respiratory viruses [47]. Therefore, we reasoned that species susceptible to respiratory pathogens might lack these ciliary genes, contributing to their higher susceptibility. Our search of approximately 500 vertebrate genomes revealed the independent loss of *WDR93* in several lineages of birds in contrast to its widespread conservation across vertebrate species from seven taxonomic classes spanning 73 orders, including mammals, birds, non-avian reptiles, amphibians and fishes. Interestingly, *CFAP46*, another C1d gene directly interacting with *WDR93,* is concurrently lost in multiple phylogenetically independent bird lineages through segmental deletions.

## 2. Methods

### 2.1. Identification of conserved one-to-one orthologues in vertebrates

We performed the comparative genomic analysis across approximately 500 vertebrate species spanning seven classes and 73 orders, consisting of species from Actinopterygii, Afrotheria, Amphibians, Artiodactyla, Aves, Carnivora, Chiroptera, Chondrichthyes, Coelacanth, Crocodilia, Insectivores, Lagomorpha, Marsupials, Monotremata, Perissodactyla, Pholidota, Primates, Rodents, Serpentes, Squamata, Testudines and Xenarthra with good-quality genome assemblies to identify one-to-one orthologues of the *WDR93* gene (see electronic supplementary material, table S1). The gene annotated as *WDR93* in the human genome was used as a reference to identify one-to-one orthologues in 23 representative species from various vertebrate clades based on synteny and sequence similarity (see electronic supplementary material, table S2). To overcome the heterogeneous annotation quality in different species, we re-annotated the *WDR93* gene using the gene sequence of the representative species in each clade when the National Center for Biotechnology Information (NCBI) annotation was incomplete or labelled as a low-quality protein. See electronic supplementary material, text/S1 for details of annotation curation and re-annotation. The re-annotation was done by running the TOGA (tools to infer orthologues from genome alignment) pipeline [48] on good-quality genomes. Subsequently, the assembly sequences were verified and rectified in case of errors using Illumina short-read sequencing (see electronic supplementary material, table S1).

### 2.2. Genome assembly validation using long-read sequencing

We validated the genome assembly using long-read sequencing to ensure the micro-synteny of the focal gene/remnants and its flanking regions. High-quality long-read datasets (PacBio and Nanopore) are available for chicken (see electronic supplementary material, table S3). We used the Burrows–Wheeler aligner (BWA) read aligner to map the long-read data to the genome of the chicken version GRCg6a (Genome Reference Consortium Chicken Build 6a). Subsequently, we retained PacBio reads greater than 20 kb, and Nanopore reads greater than 80 kb, spanning the genomic region containing the remnants of the focal gene along with flanking genes on both sides. The long-reads span the flanking genes covering the remnants of genomic features (exon, intron and UTR) of the *WDR93* gene. However, for the *CFAP46* gene, exonic remnants were not found at the syntenic locus despite conserved gene order on both sides. Consistent lack of genomic regions homologous to the *CFAP46* locus in various versions of the chicken genome assemblies (GRCg6a (RJF), GRCg7b (maternal broiler), GCA_024206055.2 (huxu breed)), long-read (PacBio and Nanopore) and short-read (Illumina) datasets suggests the loss of *CFAP46* due to a large segmental deletion. See electronic supplementary material, text/S2 for details of segmental deletion validation and estimating the size of the region lost.

### 2.3. Reconstructing the history of gene loss events

The high-quality chicken genome and long-read sequencing datasets allowed for detailed validation of gene loss. However, other galloanseriform species lack similar quality of genomic data. Hence, reconstructing the chronology of events relies upon genome assemblies and their confirmation using short-read Illumina sequencing. First, we detected the gene-disrupting events in each species using the TOGA pipeline with the ostrich (*Struthio camelus*) as a reference species. Subsequently, we verified the events using blastn with mallard (*Anas platyrhynchos*) and white-crested guan (*Penelope pileata*) as query species (see electronic supplementary material, table S4). Gene-disrupting events such as frameshift insertion/deletion, substitution leading to stop codon, exon deletion and splice-site change were detected by TOGA. Each event was confirmed based on a blastn search of the short-read Illumina data (see electronic supplementary material, table S5) and visualized using the MView utility.

### 2.4. Comparison of the transcriptional level at *WDR93* gene locus

The *WDR93* gene is highly expressed in the gonads [49] and cerebellum tissues. Hence, we screened the gonadal transcriptomes of the Chinese alligator (*Alligator sinensis)*, green anole *(Anolis carolinesis*), ostrich *(Struthio camelus*: cerebellum tissue RNA-seq data used instead of low-quality gonad data), emu (*Dromaius novaehollandiae*), zebra finch (*Taenipygia guttata*), great tit (*Parus major*), mallard (*Anas platyrhynchos*), swan goose *(Anser cygnoides)*, muscovy duck *(Cairina moschata)*, chicken *(Gallus gallus)*, turkey (*Meleagris gallopava*) and common pheasant (*Phasianus colchicus*) to compare transcription at the *WDR93* gene locus. The RNA-seq reads from public datasets (see electronic supplementary material, table S6) were mapped to the respective species reference genomes using STAR read mapper [50]. The flanking genes (*MINAR1* and *PEX11A*) are positive controls within the species, and the same tissue was considered between species. Furthermore, to verify the possibility of *WDR93* gene expression in other chicken tissues or sex-specific gene expression, we screened 67 RNA-seq datasets from various tissues (see electronic supplementary material, table S6). The transcriptional status was assessed visually using the integrative genomics viewer (IGV) browser [51]. Additionally, to overcome the restrictions of window size in IGV screenshots, the coverage of the *WDR93* gene locus and its flanking genes was calculated using the bedtools [52] genomecov command with and without split option and saved as a bedgraph file and visualized along with the exon locations and flanking genes.

### 2.5. Estimating selection strength and GC-biased gene conversion

We generated multiple sequence alignments of *WDR93* open reading frames (ORFs) for each clade using Guidance (v. 2.02) with PRANK as the aligner [53]. Each clade was assessed for sequence saturation using the index to measure substitution saturation (Iss) proposed by Xia *et al*. [54] and available in the DAMBE program. Time-calibrated phylogenetic species trees were downloaded for each clade from the TimeTree website [55,56]. We estimated selection strength in each species using RELAX program from the HyPhy package [57] and codeML from the PAML package [58] (see electronic supplementary material, tables S7 and S8). We also quantified the magnitude of gBGC using the mapNH program from testNH (v. 1.3.0) and phastBias from PHAST (v. 1.6) (see electronic supplementary material, table S9). See our earlier study [59] for detailed methods.

## 3. Results

### 3.1. Conservation of micro-synteny at *WDR93* locus

Comparison of gene order in the flanking regions of the *WDR93* gene in 23 representative species of major clades identified two main types of micro-synteny (electronic supplementary material, figure S1). All therian genomes analysed, including humans, contain *PEX11A, PLIN1* and *KIF7* on the left flank, with *MESP1*, *MESP2* and *ANPEP* genes on the right flank of *WDR93* (see electronic supplementary material, table S2). Among monotremes, while the gene order on the left flank was the same as in other mammals, the gene order on the right flank consists of *AKAP13*, *SV2B* and *SLCO3A1* genes in the platypus (*Ornithorhynchus anatinus*) and *NTRK3*, *AGBL1*, *AKAP13*, *SV2B* and *SLCO3A1* genes in the short-beaked echidna (*Tachyglossus aculeatus*) (see electronic supplementary material, table S2). The mammal-like gene order occurs in the western clawed frog (*Xenopus tropicalis*) and coelacanth (*Latimeria chalumnae*). In the case of sauropsids (including Aves), the analysed genomes had the same left flank genes as mammals, with *MINAR1*, *MTHFS* and *BCL2A1* being on the right flank of *WDR93*. The conserved synteny at the *WDR93* locus ensures unambiguous identification of 1-to-1 orthologues. Although *WDR93* shares the IPR006885 (NADH_UbQ_FeS_4_mit-like) domain with its only documented paralogue *NDUFS4*, sequence clustering analysis identified high levels of divergence between the paralogues (electronic supplementary material, figure S2).

### 3.2. Chronology of *WDR93* gene inactivation in galliformes and geese

**Figure 1.**
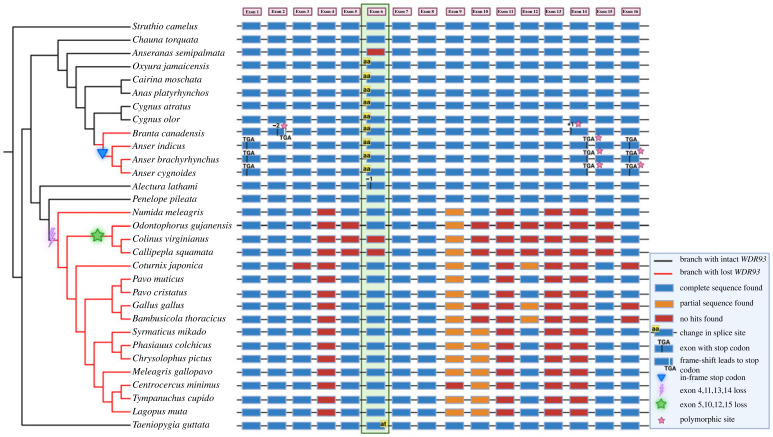
Loss of *WDR93* gene in galliform and geese species. Loss of *WDR93* in 16 galliform species, four geese species, and their phylogenetic relationship (obtained from the TimeTree website [56]) help reconstruct the chronology of events. Black and red branches indicate species with functional and pseudogenized *WDR93,* respectively. The purple thunderbolt symbol depicts a shared loss of exons 4, 11, 13 and 14 in 16 galliform species. The green star symbol represents the shared loss of exons 5, 10, 12 and 15. The blue inverted triangle represents the presence of an in-frame stop codon in the Anser lineage. The filled rectangular boxes represent exons, and the lines connecting them represent the introns. The blue boxes represent the intact exon sequences, the orange boxes represent the exons with partial sequences, and the red boxes represent the exons with no detectable remnants. The region highlighted with green represents exon 6, which has a change in the splice site (at the start of exon 6) in nine of the anseriform species. The pink star symbols indicate that the represented events are polymorphic.

None of the galliform genome assemblies contain an annotation in NCBI for the *WDR93* gene despite being well annotated in the closely related anseriform birds. Using long-read sequencing, we ruled out the possibility of assembly errors at the *WDR93* locus in the chicken genome. Three overlapping Nanopore reads greater than 100 kb (SRR15421344.63125, SRR15421342.280842 and SRR15421343.497419) span the entire region between *MINAR1* and *PEX11A* containing *WDR93* gene remnants, i.e. 5′ UTR, exons and intronic regions (electronic supplementary material, figure S3). In galliform birds, representative species from the Numididae, Odontophoridae and Phasianidae families share the deletion of four exons (exons 4, 11, 13 and 14; figure 1). Hence, the *WDR93* gene loss event shared by three of the five galliform families is estimated to have occurred between 65.4 and 46.5 Ma. Although the *WDR93* gene appears intact in the white-crested guan (*Penelope pileata*), a frame-disrupting deletion in exon 6 of the Australian brushturkey (*Alectura lathami*) suggests an independent loss (figure 1; electronic supplementary material, figure S4). The representative species from the Odontophoridae family shared the deletion of exons 5, 10, 12 and 15. The partial degradation of exons 9 and 12 was found to be shared across several galliform species. The high-quality chicken genome and long-read sequencing allowed the validation of the partially degraded exons.

Among anseriform birds, the crested screamer (*Chauna torquata*) from the Anhimidae family has an intact *WDR93* gene. The magpie goose (*Anseranas semipalmata*) is the only representative of the Anseranatidae family. The sixth exon is missing from the genome assembly of the magpie goose. Since short-read data support the validity of the magpie goose genome assembly (see electronic supplementary material, figure S5), it appears that the sixth exon is lost without causing any frameshift. All the representative species from the Anatidae family whose genomes were analysed share an AG→AA 5′ splice-site disrupting change at exon 6 (figure 1; electronic supplementary material, table S10). Independent GT→AT 3′ splice-site disrupting changes at exon 6 also occur in Neoaves species such as zebra finch (*Taeniopygia guttata*). Hence, exon 6 appears dispensable, and its loss does not result in gene loss. Duck and swan species such as the ruddy duck (*Oxyura jamaicensis*), muscovy duck (*Cairina moschata*), mallard (*Anas platyrhynchos*), black swan (*Cygnus atratus*) and mute swan (*Cygnus olor*) have intact ORFs after excluding exon 6. However, geese species from the genera Anser and Branta have gene-disrupting changes. The bar-headed goose (*Anser indicus*), pink-footed goose (*Anser brachyrhynchus*) and swan goose (*Anser cygnoides*) share a C to T substitution at the 69th base of exon 1, which leads to a premature stop codon (CGA → TGA). Additionally, the three species from the *Anser* genus share a premature stop codon (CGA → TGA) inducing polymorphism at the 154th base of exon 14, and another polymorphic site (CAT → TGA) occurs at the 62–64th position of exon 16 (see electronic supplementary material, figure S6). The genome assembly of swan goose (*Anser cygnoides*) was verified using PacBio long-read sequencing data (see electronic supplementary material, figure S7). Similarly, two gene-disrupting polymorphisms, a two-base deletion (in exon 2) and one base insertion (in exon 14), occur in the Canada goose (*Branta canadensis*), resulting in a premature stop codon in exon 2 (electronic supplementary material, figure S8).

### 3.3. Contrasting transcriptional patterns at the *WDR93* locus

**Figure 2.**
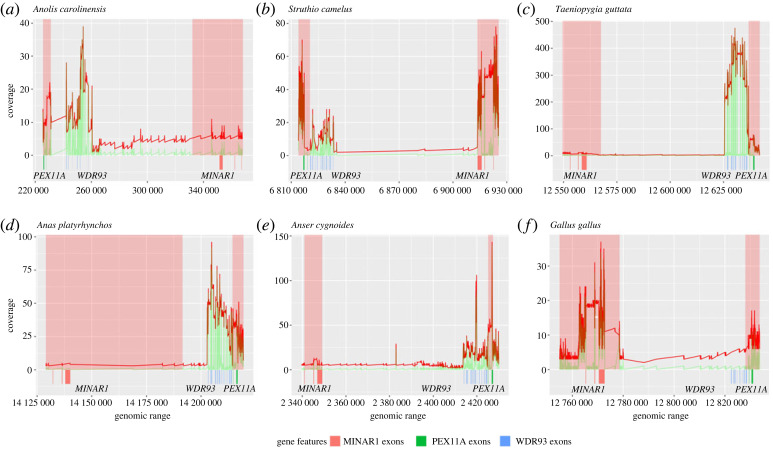
Transcriptional status of *WDR93*. Read coverage of RNA-seq data from NCBI Short Read Archive (SRA) database mapped to its corresponding species genome using STAR mapper. (*a*) Green anole (*Anolis carolinensis*), (*b*) common ostrich (*Struthio camelus*), (*c*) zebra finch (*Taeniopygia guttata*), (*d*) mallard (*Anas platyrhynchos*), (*e*) swan goose (*Anser cygnoides*), (*f*) chicken (*Gallus gallus*). The genes adjoining *WDR93*, i.e. *PEX11A* and *MINAR1,* are highlighted with a red background as a positive control. The red line in the graph shows the total RNA-seq coverage (without the split option in bedtools), and the green line indicates the spliced alignment coverage (with split). The blast hits of *MINAR1*, *PEX11A* and *WDR93* exons to the genome of corresponding species are shown in red, green and blue, respectively. RNA-seq short read data are from the testis for all species except for using cerebellum in ostrich.

The annotation of the *WDR93* gene consists of 16 exons in most vertebrate species. Examination of the transcriptomes confirmed that all 16 exons are expressed in Chinese alligator (*Alligator sinensis*), green anole (*Anolis carolinensis*), ostrich (*Struthio camelus*) and emu (*Dromaius novaehollandiae*) (figure 2; electronic supplementary material, figures S9–S16). However, as noted in the previous section, some bird species have splice-site disrupting changes in exon 6. Accordingly, only 15 exons are expressed in mallard (*Anas platyrhynchos*), muscovy duck (*Cairina moschata*), great tit (*Parus major*) and zebra finch (*Taeniopygia guttata*) (electronic supplementary material, figures S17–S24). Therefore, the RNA-seq data confirm the dispensability of exon 6 and its exclusion from the transcript in species with splice-site disrupting changes. The *WDR93* gene is prominently expressed in the gonads and cerebellum (figure 2; electronic supplementary material, figure S25). Galliform species with gene-inactivating mutations, such as chicken (*Gallus gallus*), turkey (*Meleagris gallopava*), helmeted guineafowl *(Numida meleagris)*, common pheasant (*Phasianus colchicus*), Indian peafowl (*Pavo cristatus*) and Japanese quail (*Coturnix japonica*) lack transcript read support at the *WDR93* locus in the gonad (see electronic supplementary material, figures S26–S29). However, at the same time, *WDR93* is abundantly expressed in the gonad of anseriform species such as mallard (*Anas platyrhynchos*) and muscovy duck (*Cairina moschata*). Additional screening of 67 RNA-seq datasets of various chicken tissues from male and female individuals failed to identify transcripts at the *WDR93* locus. Thus, several lines of evidence support the loss of the *WDR93* gene in chickens. Although the swan goose (*Anser cygnoides*) has gene-disrupting changes, we found RNA-seq reads mapped to *WDR93* in the gonad sample (figure 2). Upon closer inspection of the *WDR93* locus in the swan goose, the transcript support was found to be noisy, and the concordance of spliced reads with the exon boundaries was weak (see electronic supplementary material, figure S25).

### 3.4. Independent losses of *WDR93* in other Neognathae species

**Figure 3.**
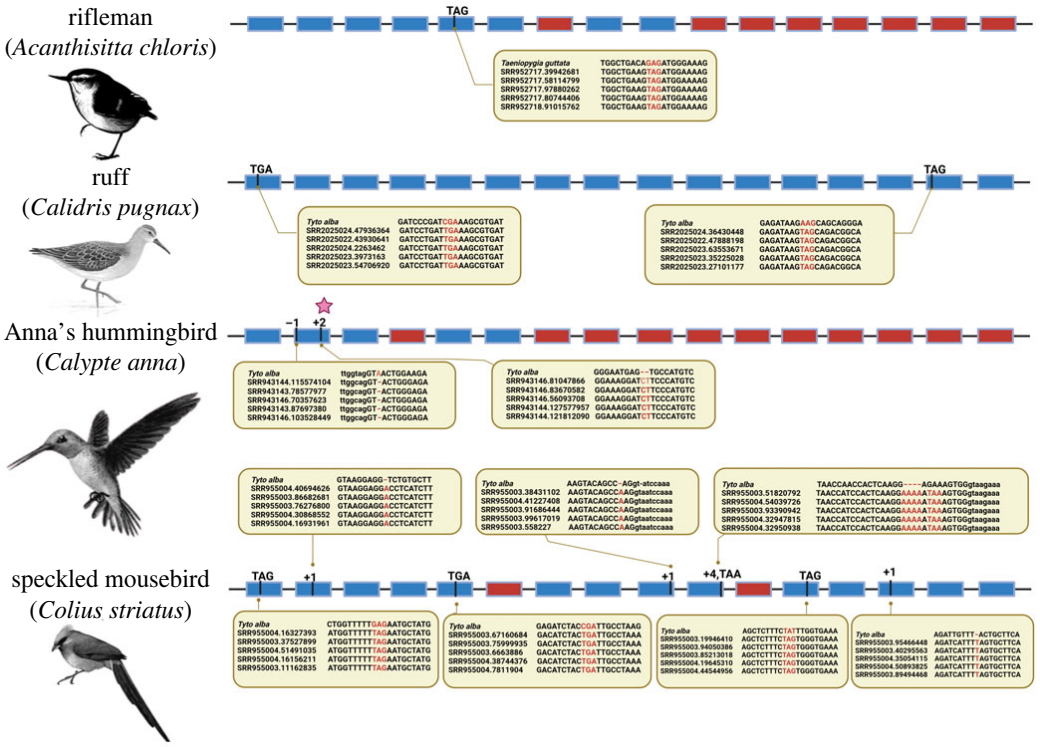
Independent loss of *WDR93* gene in Neoaves. The *WDR93* gene is lost independently in the rifleman (*Acanthisitta chloris*), ruff (*Calidris pugnax*), Anna's hummingbird (*Calypte anna*) and speckled mousebird (*Colius striatus*). The filled rectangular boxes represent exons, and the lines connecting them represent the introns. The blue ones represent the exon sequences, and the red boxes represent the exons with no remnants. The pseudogenization events are shown on top of exons with black lines and events at the corresponding locations. The inset at events, i.e. in the comment box, depict the raw read support for that event visualized using Mview. The bird images are adapted from photos available in the public domain.

The *WDR93* locus genome assembly verification and annotation step (see Methods) helped identify putative gene loss events and rectify errors. We could verify four promising examples of independent losses of *WDR93* in Neognathae (figure 3). (i) The fifth exon of *WDR93* in the rifleman (*Acanthisitta chloris*) bird has a G to T nucleotide substitution at its 57th base, leading to an in-frame stop codon (GAG → TAG). Short-read sequencing supports the validity of this substitution (figure 3). Exon 7 and exons 10–16 are all missing from the rifleman genome assembly and short-read data. The validity of the genome assembly at the *WDR93* locus was ascertained using long-read sequencing and confirms the loss of 8 of the 16 exons (see electronic supplementary material, figure S30). (ii) In the ruff (*Calidris pugnax*), exon 1 at the 103rd base and exon 15 at the ninth base have in-frame stop codons due to CGA → TGA and AAG → TAG substitutions, respectively. Short-read data support the validity of these stop codon-inducing substitutions (figure 3). (iii) Anna's hummingbird (*Calypte anna*) has one frameshifting deletion and another polymorphic frameshifting insertion in exon 2 (see electronic supplementary material, figure S31). Exon 4 and exons 7–16 are deleted in Anna's hummingbird, and the correctness of the genome assembly could be verified by a tiling path of long-read data (see electronic supplementary material, figure S32). (iv) In speckled mousebird (*Colius striatus*), the *WDR93* gene has four in-frame stop codons, three single base insertions (in exons 2, 9 and 14), and a four-base insertion in exon 10. Two exons (6 and 11) are missing from the speckled mousebird genome assembly, and short-read data suggest their loss.

### 3.5. Concurrent loss of *CFAP46* through large segmental deletions

**Figure 4.**
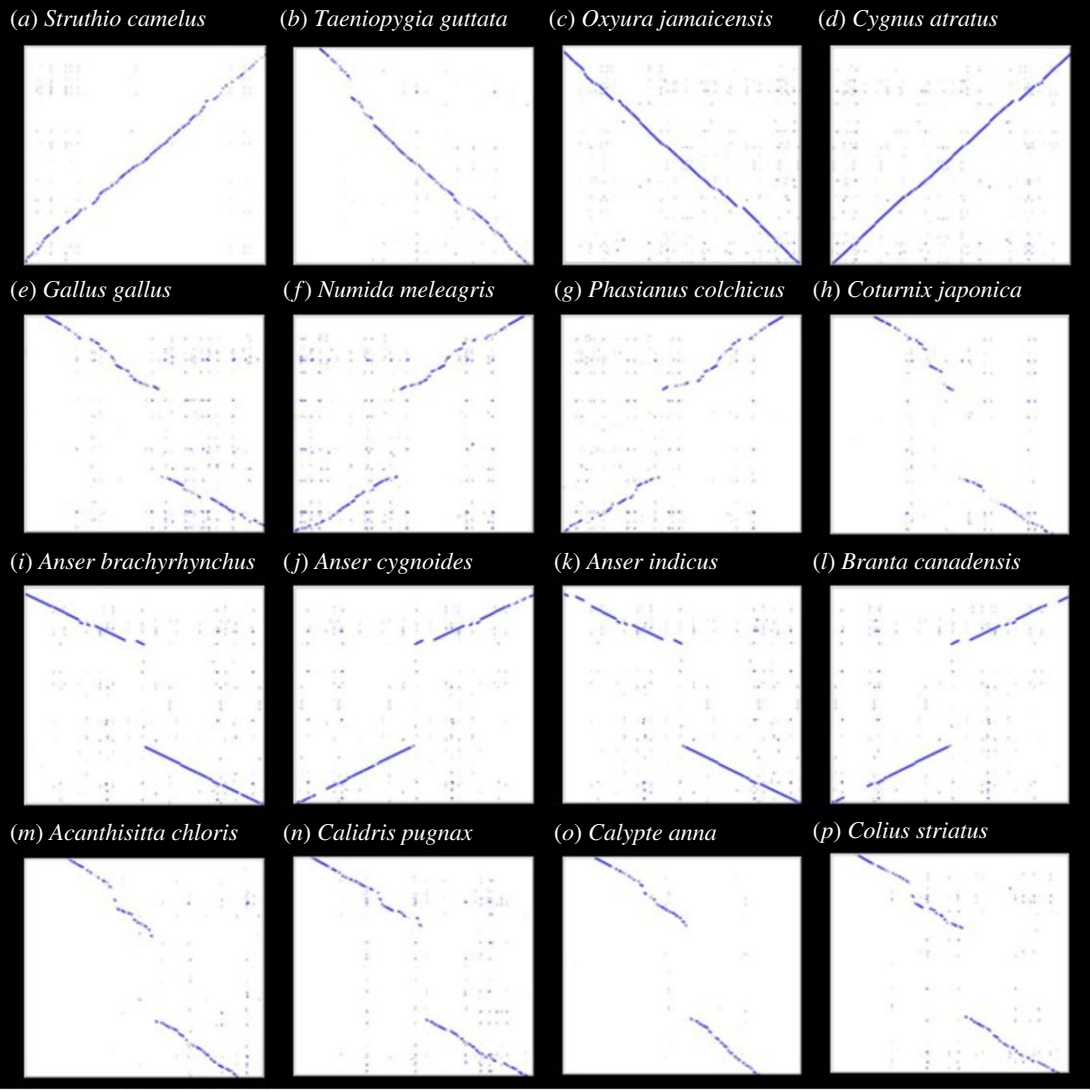
Concurrent loss of *CFAP46* gene with *WDR93*. A blast-based search of genomes of representative species (shown on the *x*-axis of each dot plot) using the entire *CFAP46* genic region, along with the 50 kb flanking sequence of the mallard (*Anas platyrhynchos*) as a query (shown on the *y*-axis of each dot plot), was used to generate dot plots using the xsltproc utility from XSLT sandbox (https://github.com/lindenb/xslt-sandbox). Conserved orthologous regions with no segmental deletions are found in (*a*) ostrich (*Struthio camelus*), (*b*) zebra finch (*Taeniopygia guttata*), (*c*) ruddy duck (*Oxyura jamaicensis*) and (*d*) black swan (*Cygnus atratus*). However, alignment patterns consistent with a segmental deletion were found in the galliform lineage in species such as (*e*) chicken (*Gallus gallus*), (*f*) helmeted guineafowl (*Numida meleagris*), (*g*) common pheasant (*Phasianus colchicus*) and (*h*) Japanese quail (*Coturnix japonica*). Similarly, in geese lineage, another segmental deletion is common to species such as (*i*) pink-footed goose (*Anser brachyrhynchus*), (*j*) swan goose (*Anser cygnoides*), (*k*) bar-headed goose (*Anser indicus*) and (*l*) Canada goose (*Branta canadensis*). Other phylogenetically independent bird species, namely (*m*) rifleman (*Acanthisitta chloris*), (*n*) ruff (*Calidris pugnax*), (*o*) Anna's hummingbird (*Calypte anna*) and (*p*) speckled mousebird (*Colius striatus*) have lost the *CFAP46* gene through segmental deletions.

The C1d projection consists of proteins encoded by *WDR93* and other genes such as *CFAP221*, *CAM1*, *CFAP54* and *CFAP46*. A search for orthologues of these genes revealed that the *CFAP46* gene-containing region is conserved in representative bird species, such as ostrich (*Struthio camelus*), zebra finch (*Taeniopygia guttata*), mallard (*Anas platyrhynchos*: used as the query) tufted duck (*Aythya fuligula*), black swan (*Cygnus atratus*), mute swan (*Cygnus olor*) and ruddy duck (*Oxyura jamaicensis*) (figure 4*a–d*). The *CFAP46* gene-containing region is missing in the chicken genome assembly. The micro-synteny of the *CFAP46* gene is conserved across birds, with *ALDH18A1* and *TCTN3* on the left flank and *OPNVA*, *NKX6–2* and *INPP5A* genes on the right flank (see electronic supplementary material, table S11). Based on pairwise genome alignment between mallard (*Anas platyrhynchos*) and chicken (*Gallus gallus*), we inferred a chicken-specific deletion containing the *CFAP46* gene. The *CFAP46* deletion is shared by other galliform species, such as turkey (*Numida meleagris*), common pheasant (*Phasianus colchicus*) and Japanese quail (*Coturnix japonica*) (figure 4*e–h*). We approximated the size of segmental deletion in various galliform species (see electronic supplementary material, table S12) to range from approximately 81 kb to approximately 73 kb. We confirmed the correctness of the genome assembly using PacBio long-read sequencing in chicken (*Gallus gallus*) and mallard (*Anas platyrhynchos*) (see electronic supplementary material, figures S33 and S34). Hence, the large segmental deletion identified in chicken is not an assembly error.

We found an independent segmental deletion of *CFAP46* in the Canada goose (*Branta canadensis*), a species in which the *WDR93* gene has gene-inactivating polymorphisms. The segmental deletion found in the Canada goose (*Branta canadensis*) also occurs in the pink-footed goose (*Anser brachyrhynchus*), swan goose (*Anser cygnoides*) and bar-headed goose (*Anser indicus*) (figure 4*i–l*). While the boundaries of the segmental deletion occur in the same region as chicken, they are not identical (see electronic supplementary material, figures S35 and S36). For instance, the size of the geese-specific deletion was approximately 92 kb (see electronic supplementary material, table S12). To ensure this region is not prone to assembly errors, we verified the genome assembly of swan goose (*Anser cygnoides*) with PacBio long-read sequencing (see electronic supplementary material, figure S37). Since we found two cases of concurrent loss of *WDR93* and *CFAP46*, the genomes of other bird species that have lost the *WDR93* gene were searched for *CFAP46* orthologues. Interestingly, the *CFAP46*-containing region of the genome appears to be lost through independent segmental deletions in the four species with independent *WDR93* gene loss, i.e. rifleman (*Acanthisitta chloris*), ruff (*Calidris pugnax*), Anna's hummingbird (*Calypte anna*) and speckled mousebird (*Colius striatus*) (figure 4*m–p*). The genome assemblies of the rifleman (*Acanthisitta chloris*) and Anna's hummingbird (*Calypte anna*) could be verified using PacBio long-read sequencing (see electronic supplementary material, figures S38 and S39). Consistent support for the deleted regions in long-read datasets, the prevalence in independent genome assemblies of closely related species (figure 4*e–l*), and the lack of short-read support for the deleted region strongly support the recurrent deletion of this *CFAP46*-containing region without translocation within the somatic genome. In summary, our study establishes that several bird species have lost *WDR93* and *CFAP46* genes that are components of the C1d projection (electronic supplementary material, figure S40).

## 4. Discussion

This study establishes the recurrent and concurrent loss of *WDR93* and *CFAP46* in birds through frame-disrupting changes and segmental deletions. While the *CFAP46* gene has no known paralogues, *NDUFS4* is the only paralogue of *WDR93*. Cluster analysis of sequences (CLANS analysis suggests *WDR93* and *NDUFS4* are highly divergent. Hence, the loss of these ciliary genes is unlikely to be compensated. Comparative genomics across approximately 500 vertebrates found widespread conservation of these genes compared with gene loss events in birds. Both genes are C1d components and have well-established roles in ciliary motility. In addition to the gene-disrupting changes, we found a lack of gene expression in the species with gene loss in the tissues, where it is robustly expressed in other species with intact genes. In contrast to widespread conservation in mammals, the ciliary gene loss in birds could be explained by previous findings that avian influenza viruses preferentially infect ciliated airway epithelial cells due to their sialic acid linkages, whereas human influenza viruses target non-ciliated cells [19,60].

### 4.1. Role of ciliary genes in host–pathogen interactions

The central ciliary apparatus of motile cilia comprises an asymmetric pair of microtubules, C1 and C2, with interconnected distinct protein complexes known as projections [40,61]. The overall structure of the central apparatus is largely conserved despite minor species-specific differences [62,63]. The study of mutants lacking specific ciliary apparatus components suggests different roles for each projection in ensuring motility [39]. The epithelial cilia in the respiratory tract perform mucociliary clearance of pathogens as a host defence strategy [64]. For instance, mice enhance ciliary activity and mucociliary clearance when infected with the influenza A virus [65]. However, pathogens can counter almost every host defence by suppressing and evading the host immune machinery [66,67]. Tactics of the pathogens, such as down-regulating ciliogenesis regulatory genes [68], targeting ciliated epithelial cells [69] and hijacking cilia to cross the nasal epithelium barrier [70], highlight the vital role of cilia in host–pathogen interactions. The central apparatus projection D deficiency results in severe motility defects and appears to be an ideal target for pathogen-mediated disruption of cilia [36,44,71]. Changes in the expression of the *WDR93* gene upon influenza virus infection strongly indicate that respiratory pathogens may target projection D components [34]. Hence, the concurrent loss of these genes in phylogenetically independent bird species raises the question of whether host–pathogen interactions are changed, resulting in differential susceptibility.

The concurrent loss of *WDR93* and *CFAP46* in multiple independent lineages of birds, in contrast to conservation across hundreds of other vertebrate species, suggests rapid shifts in selective forces acting on these genes. Loss of genes with host defence functions may result from relaxed selection following a change in pathogen repertoire or invasion strategy [72–74]. The ongoing arms race between host and pathogen involves constantly evolving new and complex mechanisms to counter each other and can also cause gene loss [75–77]. The disruption of *WDR93* and *CFAP46* genes could also be a casualty of this arms race. For instance, the host can have an adaptive advantage in suppressing or losing these genes if pathogens hijack the cilia to counter the immune response or overcome host defences [12]. The sustained expression of *RIG-I*, interferon-stimulated genes and genes from pathways involved in ciliary functions are explicitly documented in nasal epithelial cells in response to viral RNA persistence [78]. Loss of genes could also be a consequence of such persistent activation of the immune system due to increased contact with certain pathogens. Our findings indicate a hitherto unknown role for the central apparatus projection D components in host–pathogen interaction due to their involvement in ciliary motility. Interspecies comparisons have revealed a diversity in the mucociliary transport speed among birds which may be associated with differences in the ciliary machinery [79]. Hence, understanding the impact of *WDR93* and *CFAP46* gene presence/absence across diverse species in modulating the response to invading pathogens is of immunological significance. Modifying ciliary function by targeting conditionally dispensable genes such as *WDR93* and *CFAP46* could also be a promising strategy for developing new antiviral therapies.

### 4.2. Comparative study of gene loss patterns and zoonosis

Comparative studies of non-traditional animal models have great potential in understanding host-specific immune responses and developing new approaches to counter zoonotic pathogens [80–83]. However, restricted access to wild animals and the non-availability of molecular biology tools for non-model species have been major hurdles to experimentally studying exotic pathogens. Due to seasonal variations in microbial load, many ‘missing viruses’ and ‘unknown zoonoses’ are yet to be discovered and can act as potential sources of infection in the future [84–86]. Hence, novel approaches, such as those which leverage machine learning approaches for identifying potential reservoir species, intermediate hosts and susceptible species, are required [87–89]. The availability of high-quality genomic datasets has enabled large-scale comparative genomic studies that will reveal genetic differences associated with combating various pathogens and help identify potential reservoirs and species at zoonotic risk [29,90,91]. Recent developments such as telomere-to-telomere genome assemblies and transgenics have made the chicken an amenable model to compare with the mammalian immune system [92,93]. Differences between the natural immune system versus the transmission or spillover host could help better understand the host–pathogen interactions [94–96].

The bird species in which the *WDR93* and *CFAP46* genes are lost chiefly belong to the superorder Galloanserae. Our study finds ciliary gene loss in geese (which are tolerant carriers but have sporadic mortality to avian influenza virus (AIV)) in contrast to transcriptionally active intact genes in ducks (which are resistant to most AIV) [97–99]. Similarly, galliform birds (highly susceptible to AIV with high mortality) appear to have a more evolutionarily widespread loss of these ciliary genes than anseriform species. While this fits the general pattern of chicken-specific loss of immune genes, the reason remains debatable [30,100,101]. Recent studies investigating the transcriptional response to viral infection in birds reveal a complex scenario of alternative pathways that can compensate for lost genes [31,102–105]. Nonetheless, the *WDR93* and *CFAP46* gene disruption patterns suggest their loss could have functional significance for influenza infection.

The gene loss in the ruff (*Calidris pugnax*) is consistent with the Charadriiformes order being the second most important maintenance host after anseriform birds [97,106–109]. Although the role of non-Anseriformes and non-Charadriiformes (NANC) species in acting as a reservoir for the influenza virus is difficult to evaluate using current monitoring practices, gamebirds have received considerable attention [110–113]. Nonetheless, two more species with gene loss, the Anna's hummingbird (*Calypte anna*) and the rifleman (*Acanthisitta chloris*), can be infected by the avian poxvirus and the zoonotic bacterium *Chlamydia psittaci*, respectively [114–117]. The rifleman is non-migratory and endemic to New Zealand but may show severity upon pathogenic infection spread by migratory birds. The spread of the virus to other wild birds can threaten endangered species and complicate pathogen containment efforts. The loss of *WDR93* and *CFAP46* in speckled mousebird (*Colius striatus*), a species reportedly missing the *MDA5* gene [118], may indicate a different host–pathogen interaction dynamic. Identifying NANC species lacking vital immune genes could help prioritize monitoring efforts and develop more sophisticated zoonotic potential evaluation strategies.

Our detailed analysis suggests that loss of the ciliary genes *WDR93* and *CFAP46* could compromise ciliary motility in important tissues such as the respiratory epithelium, impairing mucociliary clearance. Evidence from transcriptome studies of the host response to influenza infection supports the role of *WDR93* in countering respiratory pathogens [34]. Therefore, our results suggest that the loss of these ciliary genes can be relevant to how these species deal with respiratory pathogens and explain their higher susceptibility.

## 5. Conclusion

Lineage-specific loss of widely conserved genes resulting in phenotypic consequences serves as a natural experiment to understand the function of genes. Our work provides an example of how gene presence/absence patterns can identify novel candidates, even among understudied proteins involved in processes that are experimentally difficult to test. We found the loss of ciliary genes (*WDR93* and *CFAP46*) in phylogenetically distant bird species known to be more susceptible to influenza virus infection than species with intact genes. Further work is needed to illuminate how cilia function without these genes and how specifically host–pathogen dynamics are affected. The approach outlined here also provides a framework for identifying other genes of potential relevance to host–pathogen interaction. Genome-wide extension of our study will help us understand the genomic factors involved in zoonotic spillover and find species that can act as potential reservoirs and species at zoonotic risk. We hope this approach will help in developing new strategies for disease management in the future.

## Data Availability

Data and relevant code for this research work are stored in GitHub: https://github.com/CEGLAB-Buddhabhushan/WDR93_CFAP46 and have been archived within the Zenodo repository: https://doi.org/10.5281/zenodo.8192181 [119]. The data are provided in electronic supplementary material [120].
